# Transient low T3 syndrome in patients with COVID-19: a new window for prediction of disease severity

**DOI:** 10.3389/fendo.2023.1154007

**Published:** 2023-07-13

**Authors:** Mingyao Zhong, Yue Gao, Hongling Hu, Xuan Zhu, Lulu Gan, Ling Li, Cheng Xiang, Yimin Yan, Zhe Dai

**Affiliations:** ^1^ Department of Endocrinology, Xiaogan Hospital Affiliated to Wuhan University of Science and Technology, The Central Hospital of Xiaogan, Xiaogan, Hubei, China; ^2^ Department of Internal Medicine, Medical College of Wuhan University of Science and Technology, Wuhan, China; ^3^ Department of Endocrinology, Zhongnan Hospital of Wuhan University, Wuhan, China

**Keywords:** low T3 syndrome, thyroid, COVID-19, critical illness, prediction

## Abstract

**Objective:**

To investigate the relationship of low T3 syndrome with disease severity in patients with COVID-19.

**Methods:**

The clinical data of 145 patients with COVID-19 were retrospectively collected, and patients were divided into a low T3 group and a normal T3 group. Logistic regression models were used to assess predictive performance of FT3. Receiver operating characteristic (ROC) analysis was used to evaluate the use of low T3 syndrome in predicting critical disease. Kaplan-Meier analysis was used to analyze the impact of low T3 syndrome on mortality.

**Results:**

The prevalence of low T3 level among COVID-19 patients was 34.48%. The low T3 group was older, and had lower levels of hemoglobin, lymphocytes, prealbumin, and albumin, but higher levels of white blood cells, neutrophils, CRP, ESR, and D-dimer (all *p*<0.05). The low T3 group had greater prevalences of critical disease and mortality (all *p <*0.05). Multivariate logistic regression analysis showed that the Lymphocytes, free T3 (FT3), and D-dimer were independent risk factors for disease severity in patients with COVID-19. ROC analysis showed that FT3, lymphocyte count, and D-dimer, and all three parameters together provided reliable predictions of critical disease. Kaplan-Meier analysis showed the low T3 group had increased mortality (*p*<0.001). Six patients in the low T3 group and one patient in the normal T3 group died. All 42 patients whose T3 levels were measured after recovery had normal levels after discharge.

**Conclusion:**

Patients with COVID-19 may have transient low T3 syndrome at admission, and this may be useful for predicting critical illness.

## 1 Introduction

Many studies have demonstrated that COVID-19 is associated with functional abnormalities of the thyroid (1–3), including low T3 syndrome, subclinical hypothyroidism, and subacute thyroiditis (4, 5). The most common of these is low T3 syndrome (6), and this condition had a prevalence of 64% in one population of COVID-19 patients (7). Low T3 syndrome, also referred to as non-thyroidal illness syndrome (NTIS), is a metabolic disorder of thyroid gland caused by a non-thyroidal illness that can occur upon exposure to a variety of stressful conditions that manifest as a decreased level of T3, but normal levels of T4 and TSH. Previous studies showed that a low free T3 (FT3) level was a reliable prognostic indicator for patients with a variety of diseases. In particular, a low FT3 level is a reliable predictor for death in ICU patients (8, 9).

In November 2021, researchers first discovered the Omicron variant of SARS-CoV-2 in Botswana (10). This strain is highly transmissible (11), and 3 weeks after its discovery it replaced Delta as the predominant SARS-CoV-2 variant (12). Although Omicron is generally associated with reduced disease severity and mortality (13), it is also associated with a higher incidence of critical illness and poor prognosis in unvaccinated older adults (14). Therefore, early prediction of critical illness is particularly important. The COVID-19 diagnostic and treatment guidelines clearly indicate that Lymphopenia is an early warning indicator for severe COVID-19 (15). Many studies have shown that elevated D-dimer is associated with severe COVID-19 (16). The value of low T3 syndrome in predicting the prognosis of COVID-19 patients remains unclear. Therefore, we retrospectively examined the predictive value of low T3 syndrome in patients who were critically ill with COVID-19.

## 2 Patients and methods

### 2.1 Patients

**Figure 1:**
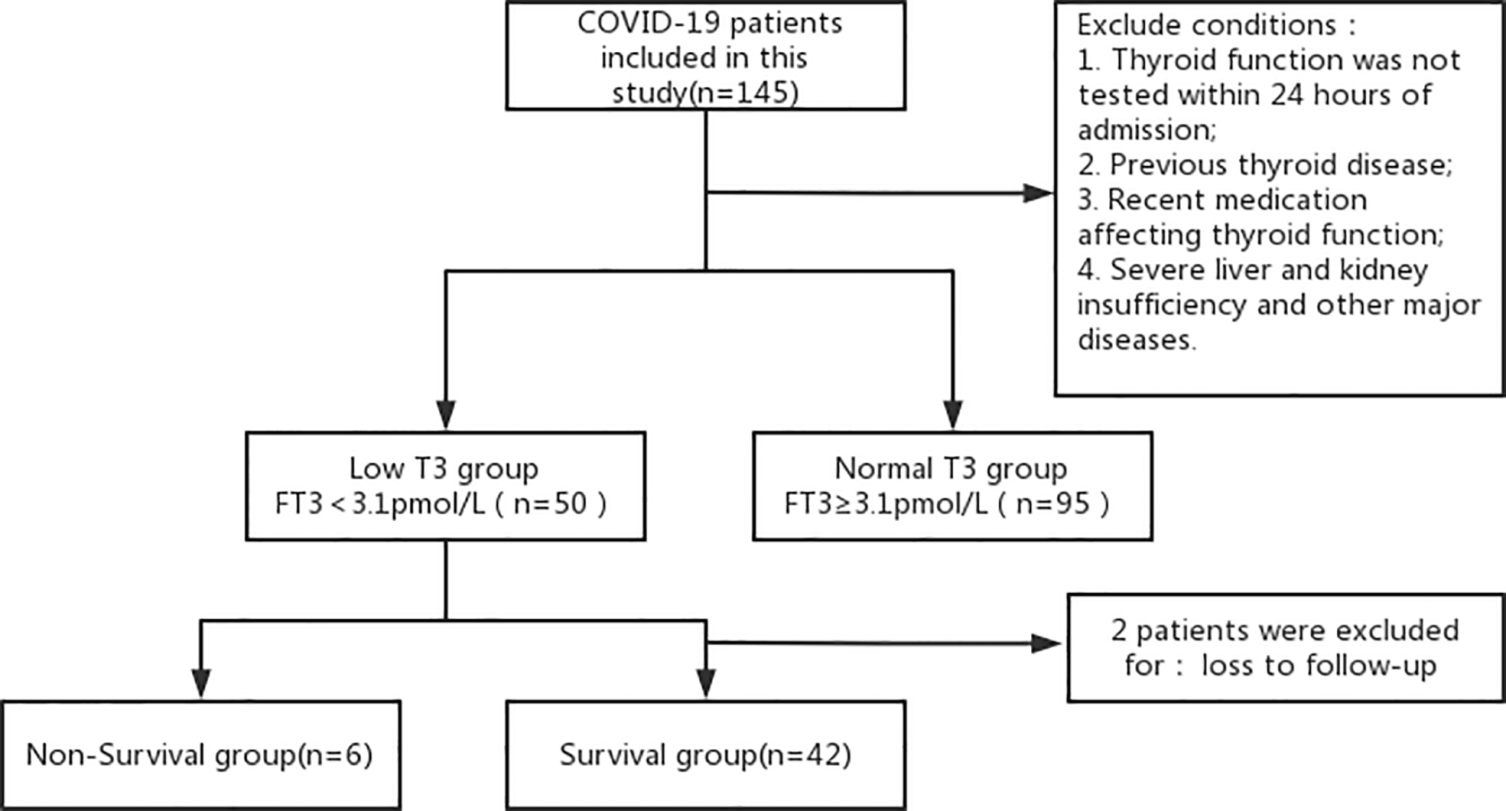
Disposition of patients who were admitted for COVID-19 and received thyroid hormone testing.

The clinical data of 145 patients diagnosed with COVID-19 from January to March 2020 in Xiaogan Central Hospital (Hubei Province) were retrospectively analyzed and divided into a low T3 group (FT3 < 3.1 pmol/L, n = 50) and a normal T3 group (FT3 ≥ 3.1 pmol/L, n = 95) (**Figure 1**). All clinical parameters were recorded within 24 h after admission. Disease severity was classified according to clinical symptoms and chest imaging results as: mild (mild symptoms, with no imaging features of pneumonia), ordinary (symptoms of fever and cough, with imaging features of pneumonia), severe (dyspnea with respiratory rate of 30/min or more, blood oxygen saturation of 93% or less, partial pressure ratio of arterial oxygen to inspired oxygen [PaO_2_/FiO_2_] below 300 mmHg, and/or pulmonary infiltration greater than 50% within 24 to 48 h after admission) or critically ill (respiratory failure, infectious shock, and/or multi-organ dysfunction or failure). All patients signed informed consent documents. The study protocol was approved by the Ethics Committee of the Xiaogan Central Hospital and followed the ethical principles of the Declaration of Helsinki, as amended in 2013.

The inclusion criteria were receipt of thyroid function testing within 24 h after admission; age of 18 years or more; and presence of the clinical diagnostic criteria for COVID-19 based on the National Health Board COVID-19 Guidelines (version 8). The exclusion criteria were previous primary thyroid disease or another endocrine disease; recent use of medications that could affect thyroid hormone secretion and/or metabolism (including amiodarone, interferon, and glucocorticoids); exposure to iodine-containing contrast media prior to thyroid testing; and presence of a chronic disease, such as hepatic insufficiency, renal insufficiency, or heart failure; high level (≥10.0 µIU/mL) or low level (<0.1 µIU/mL) of thyroid-stimulating hormone (TSH).

### 2.2 Data collection

Demographic and clinical data of patients were extracted from the hospital records system. All data regarding clinical manifestations, disease severity, laboratory results, imaging results, and clinical outcomes were recorded. Blood samples were collected within 24 h after admission. The Roche Cobas e602 electrochemiluminescence analyzer (Roche, Germany) was used to determine the levels of FT3, FT4, and TSH. RT-PCR was used to confirm SAS-CoV-2 infection from a throat swab.

During the collection of lung CT imaging data (soon after admission), two experienced physicians read the films and performed quantitative analysis of the distribution, size, and number of lesions. All 50 patients in the low T3 group were followed up; 6 of them died and 44 survived, 42 of whom returned to the outpatient clinic for rechecking of thyroid function at 1 month after discharge. One of the 95 patients in the normal T3 group died.

### 2.3 Statistical analysis

Data were analyzed using SPSS version 22.0. A difference was considered statistically significant when the P value was below 0.05. Non-normally distributed data were expressed as medians and interquartile ranges (IQRs), and the significance of differences was determined using the Mann-Whitney U test. Categorical data were expressed as numbers and percentages, and the significance of differences was determined using the Chi-squared test or Fisher’s exact test.

To assess predictive performance of FT3 in this small sample size, we firstly performed univariable logistic regressions to select potential laboratory factors associated with COVID-19 severity. Statistically significant factors were further included into multivariable logistic regression. Area under Receiver operating characteristic curve (AUC) and 95% confidence interval (CI) were reported for each selected factor and final model.

To further explore prognostic effect of FT3, Kaplan-Meier survival curves were used to determine the relationship of low FT3 with different endpoints, and survival times and 95% confidence intervals (CIs) were reported.

## 3 Results

### 3.1 Clinical characteristics of patients with low T3 and normal T3

We retrospectively examined the records of 145 patients who had COVID-19 and received thyroid hormone testing (**Table 1**). Overall, 34.50% of the patients had low T3, 52.40% were female, and the median age was 50 years (IQR 39, 60). Comparison of the two groups indicated the low T3 group was older (median age: 57 years (48, 67) vs 45 years (35, 54), *p* < 0.001) and low SpO2 (28.00% vs 6.30%, *p* < 0.001).

**Table 1:** Clinical characteristics and outcomes of all patients, the low T3 group, and the normal T3 group.

Variable	All patients (n=145)	Low T3 (n=50)	Normal T3 (n=95)	p value
Age (years), median (IQR)	50 (39,60)	57 (48,67)	45 (35,54)	<0.001
Gender, n (%)				
Male	69 (47.59)	18 (36.00)	51 (53.68)	0.043
Female	76 (52.41)	32 (64.00)	44 (46.32)	0.043
Clinical parameters on presentation, n (%)				
Temperature ≥ 37.3°C	47 (32.41)	15 (30.00)	32 (33.68)	0.363
SpO_2_ ≤ 93%	20 (13.79)	14 (28.00)	6 (6.32)	<0.001
Heart rate > 100 bpm	26 (17.93)	14 (28.00)	12 (12.63)	0.022
Mean arterial pressure, mmHg	94 (87,99)	91 (83,100)	95 (89,99)	0.182
Laboratory results				
FT3 (2.43-6.01pmol/L)	3.43 (2.80-4.00)	2.64 (2.41-2.80)	3.76 (3.44-4.26)	<0.001
FT4 (9.01-19.05pmol/L)	15.37 (13.45-17.19)	14.73 (13.31-16.32)	15.67 (13.76-17.24)	0.274
TSH (0.35-4.94uIU/mL)	1.65 (1.00-2.84)	1.10 (0.62-2.66)	1.90 (1.33-2.87)	0.002
White blood cells (3.5-9.5×10^9^/L)	4.56 (3.41-5.68)	5.33 (3.58-6.81)	4.43 (3.34-5.40)	0.005
Hemoglobin (130-175g/L)	131.00 (120.00-144.00)	124 (117-134.25)	138.00 (126.00-149.00)	<0.001
Neutrophils (1.8-6.3×10^9^/L)	2.84 (2.04-4.29)	4.27 (2.48-6.51)	2.47 (1.84-3.50)	<0.001
Lymphocytes (1.1-3.2×10^9^/L)	0.94 (0.66-1.42)	0.84 (0.57-1.00)	1.11 (0.72-1.54)	<0.001
Pre-albumin (200-400m g/L)	143.20 (109.00-190.90)	109.10 (75.30-143.58)	164.40 (132.30-206.70	<0.001
Total plasma protein (65-85g/L)	66.60 (63.80-73.00)	65.50 (63.03-69.85)	67.60 (64.30-73.85)	0.042
Albumin (40-55g/L)	38.10 (35.70-40.90)	35.75 (33.48-37.93)	39.80 (37.40-41.90)	<0.001
Procalcitonin (0-0.5ng/mL)	0.20 (0.15-0.34)	0.18 (0.16-0.35)	0.21 (0.14-0.34)	0.829
C-reactive protein (0-3mg/L)	17.91 (6.91-37.05)	36.91 (17.81-74.00)	14.90 (5.13-25.72)	<0.001
Erythrocyte sedimentation rate (0-15mm/h)	41.00 (22.00-57.00)	53.50 (30.50-84.75)	31.50 (21.00-47.25)	0.004
D-dimer (0-1mg/L)	0.27 (0.23-0.37)	0.36 (0.26-0.65)	0.26 (0.23-0.30)	<0.001
Disease severity, n (%)				
Ordinary	105 (72.41)	29 (58.00)	76 (80.00)	0.005
Severe	26 (17.93)	13 (26.00)	13 (13.68)	0.066
Critical	13 (8.97)	8 (16.00)	5 (5.26)	0.031
Clinical outcome, n (%)				
Discharge	138 (95.17)	44 (88.00)	94 (98.95)	0.012
Death	7 (4.83)	6 (12.00)	1 (1.05)	0.012

The two groups also had differences in many laboratory findings. In particular, the low T3 group had significantly lower levels of FT3, TSH, lymphocytes, hemoglobin, pre-albumin, total plasma protein, and albumin, and significantly higher levels of white blood cells, neutrophils, C-reactive protein (CRP), erythrocyte sedimentation rate (ESR), and D-dimer (all *P* < 0.05). Thus, the low T3 group had higher levels of inflammatory indicators and lower levels of nutritional parameters.

Analysis of disease severity showed that the low T3 group had a smaller proportion of patients with ordinary disease (58% vs 80%, *p* < 0.05), but greater proportions of patients with severe disease (26.00% vs 13.68%, *p* < 0.05) and critical disease (16.00% s 5.26%, *p* < 0.05). The low T3 group also had a lower rate of discharge (88.00% vs 98.95%, *p* < 0.05), and a greater rate of death (12.00% vs 1.05%, *p* < 0.05).

### 3.2 Univariate and multivariate logistic regression analysis on disease severity

Univariate logistic regression analysis showed that FT3, White blood cells, Neutrophils, Lymphocytes, Pre-albumin, Albumin, CRP, ESR, D-dimer were associated with disease severity. Multivariate logistic regression analysis showed that the Lymphocytes 0.87(95% CI: 0.82–0.93, P<0.001), FT3 0.80(95% CI: 0.51–0.93, P=0.043), and D-dimer 5.78(95% CI: 1.48–22.54, P=0.011) were independent risk factors for disease severity in patients with COVID-19. (**Table 2**).

**Table 2:** Univariate and multivariate logistic regression analysis on disease severity.

Variables	Univariate analysis		Multivariate analysis	
	OR (95%CI)	P Value	OR (95%CI)	P Value
FT3	0.42 (0.25-0.71)	0.001	0.80 (0.51-0.93)	0.043
TSH	0.98 (0.80-1.19)	0.817		
White blood cells	1.65 (1.33-2.03)	<0.001	1.45 (0.85-2.46)	0.171
Hemoglobin	0.99 (0.97-1.00)	0.125		
Neutrophils	1.13 (1.08-1.18)	<0.001	1.01 (0.80-1.28)	0.952
Lymphocytes	0.87 (0.82-0.91)	<0.001	0.87 (0.82-0.93)	<0.001
Pre-albumin	0.99 (0.98-1.00)	0.005	1.00 (0.98-1.01)	0.577
Total plasma protein	1.01 (0.96-1.07)	0.626		
Albumin	0.72 (0.63-0.83)	<0.001	0.91 (0.70-0.95)	0.040
CRP	1.02 (1.01-1.03)	<0.001	1.02 (1.01-1.04)	0.042
ESR	1.03 (1.01-1.05)	0.004	1.00 (0.96-1.04)	0.952
D-dimer	11.17 (2.14-58.28)	0.004	5.78 (1.48-22.54)	0.011

### 3.3 Predictive value of lymphocytes, D-dimer, and FT3 with disease severity

**Figure 2:**
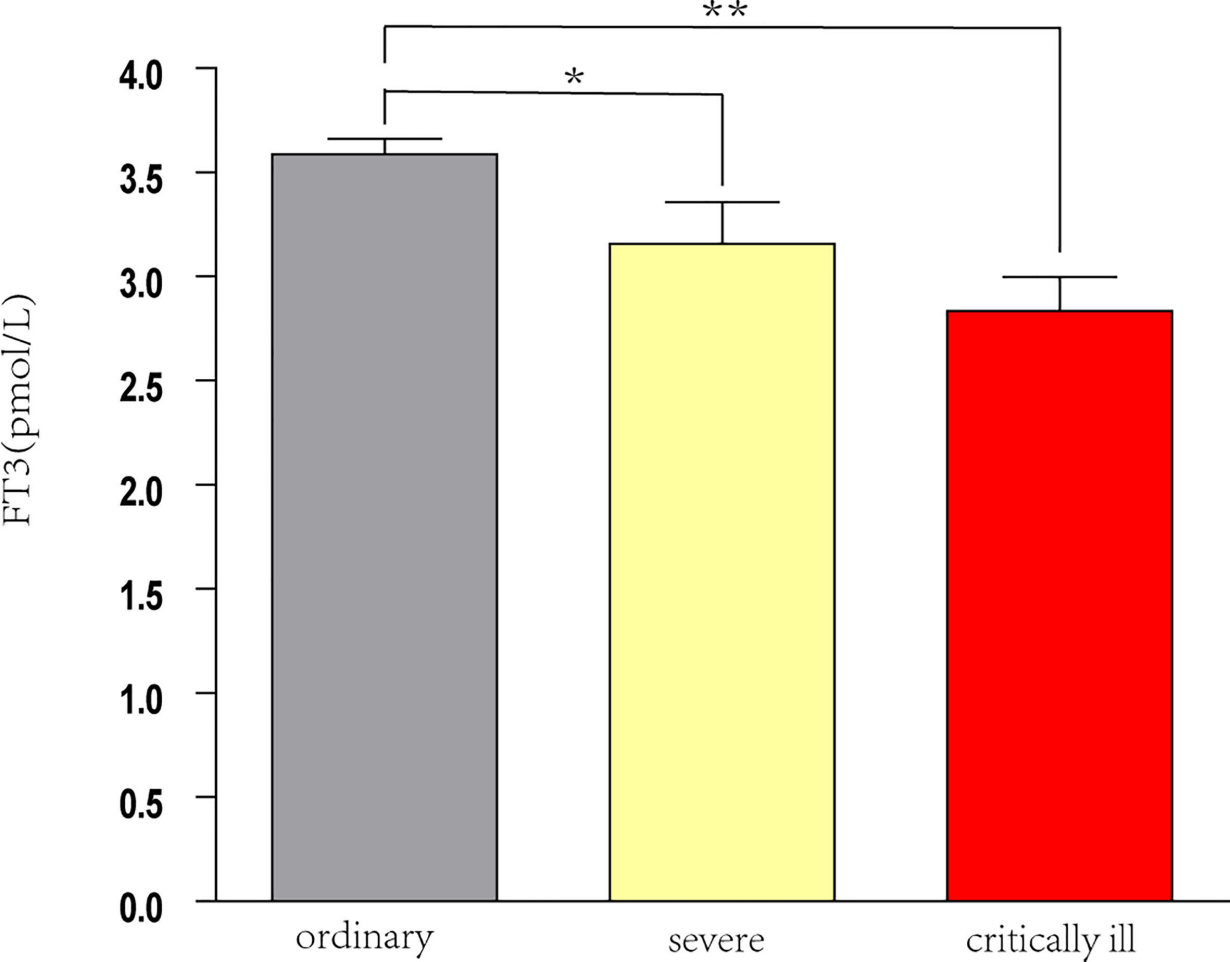
FT3 levels in patients with different severity of COVID-19. *P < 0.05, **P < 0.001.

Analysis of all 145 patients showed that as disease severity increased, the levels of FT3 and lymphocytes decreased, and the level of D-dimer increased (all *P* < 0.05; **Table 3**). Pairwise comparisons indicated the serum FT3 level was significantly lower in patients with severe disease and critical disease than in those with ordinary disease (both *p* < 0.05), but the FT3 level was not significantly different in patients with severe disease and critical illness (**Figure 2**).

**Table 3:** Levels of FT3, lymphocytes, and D-dimer in COVID-19 patients with different disease severity.

	Normal range	All (n=145)	Ordinary (n=106)	Severe (n=26)	Critically ill (n=13)	p value
FT3 (pg/mL)	3.1–6.8	3.43 (2.80,4.00)	3.59 (2.96,4.08)	3.07 (2.55,3.35)	2.89 (2.45,3.25)	0.001
Lymphocytes (×10^9^/L)	1.1–3.2	0.94 (0.66,1.42)	1.07 (0.76,1.49)	0.69 (0.52,1.17)	0.68 (0.46,0.88)	0.002
D-dimer (mg/L)	0–1	0.27 (0.23,0.37)	0.026 (0.23,0.30)	0.39 (0.25,2.21)	0.40 (0.31,1.20)	<0.001

**Figure 3:**
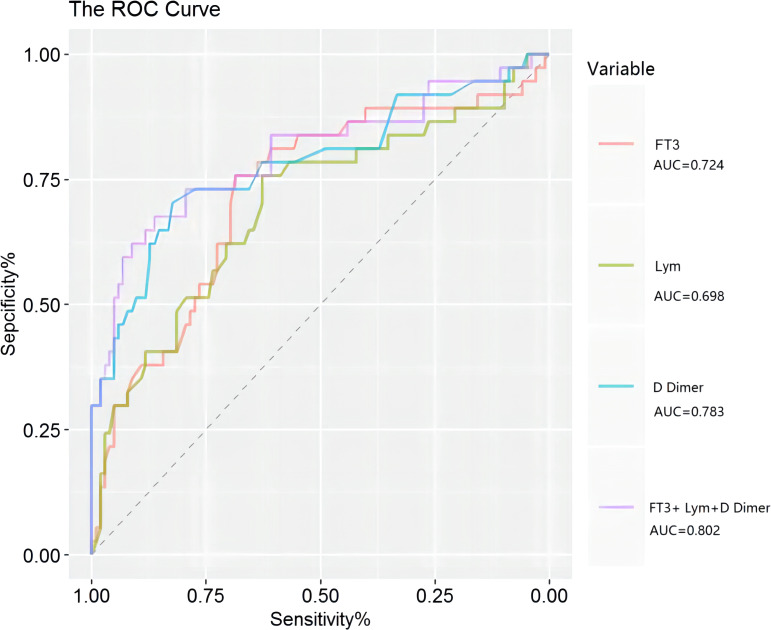
ROC curves for FT3, lymphocytes, and D-dimer in predicting critical COVID-19.

We used ROC analysis to determine the predictive performance of FT3 level, lymphocyte count, and D-dimer level on critical illness (**Table 4**, **Figure 3**). The area under the curve (AUC) was 0.724 (95% CI: 0.622–0.825, *P* < 0.001) for FT3, 0.698 (95% CI: 0.591–0.804, *P* < 0.001) for lymphocytes, and 0.783 (95% CI: 0.684–0.881, *P* < 0.001) for D-dimer. Thus, a lower FT3 level and lymphocyte count and a higher D-dimer level were significantly associated with critical illness. The combined use of all three parameters had an AUC of 0.802 (95% CI: 0.706–0.898, *P* < 0.001).

**Table 4:** Diagnostic performance of lymphocytes, D-dimer, and FT3 in predicting critical COVID-19.

Parameter	AUC	95%CI	P
FT3	0.724	0.622,0.825	<0.001
Lymphocyte count	0.698	0.591,0.804	<0.001
D-dimer	0.783	0.684,0.881	<0.001
All 3 parameters	0.802	0.706,0.898	<0.001

AUC: area under the curve.

### 3.4 Clinical characteristics and cumulative morality of patients with low T3

**Figure 4:**
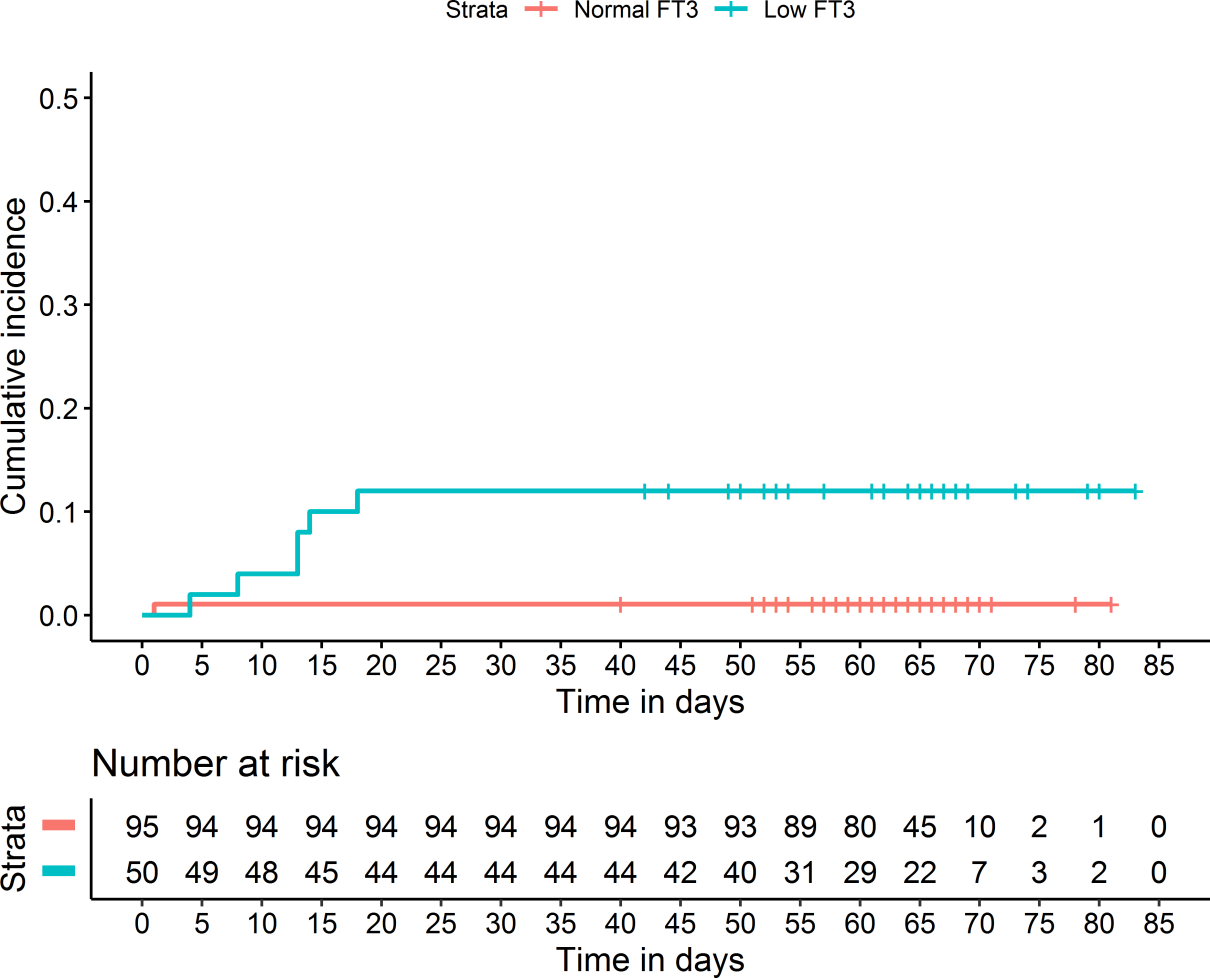
Kaplan-Meier analysis of the effect of FT3 on mortality of COVID-19 patients.

To explore the potential value of low T3 for clinical outcome assessment, we analyzed the low T3 group by comparison of survivors (n = 42) and non-survivors (n = 6; **Table 5**). The non-survivors had significantly lower levels of FT3 and lymphocytes, and a significantly higher level of D-dimer (all *p* < 0.05). Kaplan-Meier analysis confirmed that patients with low T3 had greater cumulative mortality (p < 0.001; **Figure 4**).

**Table 5:** Clinical data of patients in the low T3 group who were survivors and non-survivors.

Characteristic	Survivors (n=42)	Non-survivors (n=6)	p value
Clinical symptoms, n (%)			
Fever	40 (95.24)	5 (83.33)	0.318
Cough	28 (66.67)	6 (100.00)	0.109
Dyspnea	13 (30.95)	5 (83.33)	0.028
Fatigue	16 (38.10)	2 (33.33)	0.608
Laboratory tests, mean (IQR)			
FT3 (pmol/L)	2.68 (2.53,2.80)	2.47 (1.91,2.77)	0.034
Lymphocyte count (×10^9^/L)	0.98 (0.89,1.14)	0.68 (0.47,0.92)	0.008
Albumin (g/L)	38.70 (36.35,40.35)	33.90 (32.40,35.10)	0.073
D-dimer (mg/L)	0.25 (0.24,0.35)	0.81 (0.37,3.51)	0.035
C-reactive protein (mg/dL)	10.90 (7.79,34.45)	73.25 (39.83,137.49)	0.073
Treatments, n (%)			
Oxygen therapy	8 (19.05)	4 (66.67)	0.043
Invasive/Noninvasive mechanical ventilation	0	3 (50.00)	0.003

### 3.5 Thyroid hormone levels before and after discharge of patients with low T3

42 patients with low T3 were measured after recovery had normal levels after discharge. Analysis of the 42 patients with low T3 levels indicated these patients had significantly greater post-discharge levels of FT3 and FT4 (both *p* < 0.05; **Table 6**). The post-discharge level of TSH was also greater, but the difference was not statistically significant (*P* > 0.05).

**Table 6:** Levels of FT3, FT4, and TSH of patients in the low T3 group (n = 42) at admission and after discharge.

Measurement time	FT3 (pmol/L)	FT4 (pmol/L)	TSH (µIU/mL)
Admission	2.62 ± 0.10	17.10 ± 5.81	1.81 ± 0.18
Post-discharge	4.05 ± 0.98	17.65 ± 1.29	1.93 ± 0.21
*p* value	<0.001	0.003	0.299

## 4 Discussion

Our assessment of the prognostic value of FT3 in patients who had COVID-19 indicated that patients with low FT3 syndrome were older, had more severe clinical symptoms, and had worse clinical outcomes. We also found lower levels of FT3 and lymphocytes and a higher level of D-dimer in patients with critical illness and in non-survivors, suggesting that a low T3 level is related to COVID-19 severity. Our ROC analysis demonstrated that a low FT3 level was a reliable and independent risk factor for critical disease (AUC = 0.724), and the addition of lymphocyte count and D-dimer further improved the predictive performance (AUC = 0.802). Our analysis of the 42 patients who had low T3 syndrome and were survivors showed that thyroid function increased after recovery.

We can suggest several possible general mechanisms that explain the association between NTIS and COVID-19-related adverse outcomes. Importantly, these different mechanisms are not mutually exclusive. One possible mechanism is that the virus causes direct damage of cells in thyroid tissues. This seems plausible because angiotensin converting enzyme 2 (ACE2) functions as a receptor for the SARS-CoV-2 spike protein (17), and has high expression in thyroid tissues (18).

Second, the increased levels of cytokines induced by severe COVID-19 could explain the relationship of low T3 syndrome with poor outcome. A cytokine storm affects the course and severity of disease (19). Our low T3 group was sicker and had a stronger inflammatory response, with significantly higher levels of leukocytes, CRP, and ESR, important indicators of inflammation. This systemic inflammatory response can cause an increase in inflammatory cytokines such as IL-1, IL-6 and TNF-α (20), leading to suppression of the hypothalamic-pituitary-thyroid axis (3) and reduced 5’-monodeiodinase activity (21), resulting in decreased TSH secretion, and decreased conversion of T4 into rT3. Severe inflammation is also considered a major cause of disseminated intravascular coagulation (DIC) (22), and many COVID-19 patients have thrombosis and DIC, consistent with our finding of an elevated D-dimer level in patients with low T3.

Third, the association of a low T3 level with more severe COVID-19 disease may be because normal levels of thyroid hormones are important in protecting the lungs from injury (23). In particular, serum T3 can increase the synthesis of lung surface-active substances, reduce alveolar surface tension, and increase lung compliance, thereby improving lung function (24). In contrast, a low T3 level may lead to lower levels of lung surface-active substance and aggravate lung function in patients with COVID-19. Consistent with our findings, previous studies of patients with low FT3 and sepsis had reduced oxygen saturation, greater involvement of lung lesions, greater use of oxygen therapy, and were more likely to experience respiratory failure.

Fourth, the relationship of low T3 level with anemia with poor nutritional status may be responsible for the association of low T3 level with disease severity. Previous research reported that a low serum albumin level alone was a sufficient indicator of malnutrition in patients hospitalized with COVID-19 (25).A negative nitrogen balance and organismal depletion associated with the disease can also lead to a decrease in serum thyroid hormone transporter protein levels, inhibiting T3 production as well as T4 transport in tissues (26). Another study showed that NTIS was related to a significantly decreased level of T3 and a significantly increased level of reverse T3 (rT3, an inactive form of T3) during the acute phase of disease. This may be related to the decreased level of thyroid hormone binding protein and albumin, as well as reduced binding activity (27).

Several previous studies showed that the level of D-dimer (28) and the lymphocyte count (29) were associated with poor outcome in COVID-19 patients. Another study found that a D-dimer level of 2.0 μg/mL or more upon admission was the optimal cut-off for predicting in-hospital mortality from COVID-19 (30). Other studies reported that low T3 syndrome was strongly associated with the severity and prognosis of critical illnesses. For example, a prospective trial of 480 patients in intensive care units reported that the FT3 level was an independent and robust predictor of mortality (31). These previous studies led us to speculate that a low T3 level could be useful as a predictor of critical COVID-19, because early identification of patients who have a risk of progression to severe COVID-19 is essential for providing timely treatments. Our analysis of the low T3 group demonstrated that the level of T3 was lower in non-survivors and in those with more severe disease, and our ROC analysis demonstrated that the FT3 level was a acceptable predictor of critical COVID-19. Recent studies of COVID-19 patients also demonstrated an association between lymphopenia and thyroid function (32), indicating a potential interaction between the hypothalamic-pituitary-thyroid axis and the immune system (33). The levels of lymphocytes and D-dimer affect the relationship between COVID-19 and the thyroid, because a strong inflammatory response and coagulation dysfunction predict worse clinical outcome. In agreement, our ROC analysis indicated that using the combination of the levels of T3, lymphocytes, and D-dimer led to better prediction of critical disease.

It is possible that COVID-19 could have a long-term impact on thyroid function. However, our follow-up of 42 survivors in the low T3 group demonstrated that recovery from COVID-19 was related to recovery of thyroid function. In particular, the COVID-19 patients in the low T3 group had normalization of the levels of T3 and T4 after discharge, and all of these patients had serum T3 levels in the normal range. Some researchers suggested the use of thyroid hormone supplementation to restore the normal serum levels of patients with NTIS. However, this idea remains highly controversial and there is still no clear evidence that this supplementation provide a benefit (7, 34). Our patients experienced restoration of normal serum T3 levels after recovery from COVID-19, and none of them received thyroid hormone therapy.

There are several possible reasons why the serum T3 levels of our patients normalized after recovery from COVID-19. After disease recovery, the viral load decreases, and this could reduce the direct damage of the thyroid gland caused by SARS-CoV2. At the same time, the clearance of inflammatory cytokines from the body after recovery reduces their effect on deiodinase, thus promoting the production of T3. In addition, the negative nitrogen balance (caused by fever and inadequate nutrition during the course of the disease) normalized after recovery, and the increased serum level of protein enables the increased synthesis of thyroid hormone. The mechanism responsible for the effect of low T3 level on COVID-19 severity and the mechanism responsible for the normalization of the T3 level after recovery from COVID-19 are still unclear and need further study.

This study was limited in that it was a retrospective study of patients with COVID-19 whose thyroid function was assessed upon admission. A second limitation is that post-discharge follow-up was only performed for patients in the low T3 group, and this could have biased the results. A third limitation is that the sample size was small and all patients were from a single center. A large prospective study is needed to further examine the relationship of low T3 level with COVID-19 severity.

In conclusion, our results suggest that low T3 level is a transient injury caused by COVID-19, and is closely related to disease severity. A low T3 level was also a good predictor for critical illness, and may be useful for the early evaluation of COVID-19 patients.

## Data availability statement

The raw data supporting the conclusions of this article will be made available by the authors, without undue reservation.

## Ethics statement

The studies involving human participants were reviewed and approved by Xiaogan Central Hospital. The patients/participants provided their written informed consent to participate in this study.

## Author contributions

MZ and YG wrote the manuscript. YY and ZD conducted the design of the study and reviewed/edited the drafts, and is guarantor. XZ, LG, LL and CX collected and analyzed the data. HH revised the manuscript. All authors contributed to the article and approved the submitted article.
